# Spermatogenesis Associated Retrogenes Are Expressed in the Human Ovary and Ovarian Cancers

**DOI:** 10.1371/journal.pone.0005064

**Published:** 2009-03-31

**Authors:** Jan Rohozinski, Matthew L. Anderson, Russell E. Broaddus, Creighton L. Edwards, Colin E. Bishop

**Affiliations:** 1 Department of Obstetrics and Gynecology, Baylor College of Medicine, Houston, Texas, United States of America; 2 Institute for Regenerative Medicine, Wake Forest University, Winston-Salem, North Carolina, United States of America; 3 Department of Pathology, The University of Texas M.D. Anderson Cancer Centre, Houston, Texas, United States of America; University of Minnesota, United States of America

## Abstract

**Background:**

Ovarian cancer is the second most prevalent gynecologic cancer in women. However, it is by far the most lethal. This is generally attributed to the absence of easily detectable markers specific to ovarian cancers that can be used for early diagnosis and specific therapeutic targets.

**Methodology/Principal Findings:**

Using end point PCR we have found that a family of retrogenes, previously thought to be expressed only in the male testis during spermatogenesis in man, are also expressed in normal ovarian tissue and a large percentage of ovarian cancers. In man there are at least eleven such autosomal retrogenes, which are intronless copies of genes on the X chromosome, essential for normal spermatogenesis and expressed specifically in the human testis. We tested for the expression of five of the known retrogenes, *UTP14C*, *PGK2, RPL10L*, *RPL39L* and *UBL4B* in normal human ovary and ovarian cancers.

**Conclusions/Significance:**

We propose that the activation of the testis specific retrogenes in the ovary and ovarian cancers is of biological significance in humans. Because these retrogenes are specifically expressed in the ovary and ovarian cancers in the female they may prove useful in developing new diagnostic and/or therapeutic targets for ovarian cancer.

## Introduction

Although ovarian cancer is the second most prevalent gynecological cancer in women, it is by far the most lethal. It is the fifth leading cause of cancer-related death among women in the United States even though it represents less than 4% of the total cancers diagnosed. In 2008, it is estimated that there will be about 21,650 new cases of ovarian cancer diagnosed in the United States and there will be 15,520 attributed deaths[1]. Approximately 45% of women diagnosed with ovarian cancer will die within 5 years of diagnosis. This compares with 14% of those diagnosed with breast cancer and 30% with cancer of the cervix and uterus[2]. This high level of morbidity is thought to be due to an inability to recognize the presence of early stage ovarian cancer in the clinical setting due to the lack of cancer specific markers that could aid early diagnosis and post operative monitoring for disease recurrence. This situation is clearly illustrated by the fact that when ovarian cancers are diagnosed at stage I and II 82% of victims survive five years after diagnosis whereas those diagnosed at stage III and IV have a five year survival rate of 25%. Only 32% of cases are diagnosed at stage I and II, compared with 68% at stage III and IV[3]. There is therefore a great need to identify patterns of gene expression that are specific to ovarian cancer so that new diagnostic and therapeutic strategies can be developed.

As part of an ongoing program studying the role of genes critical for spermatogenesis, we previously identified a retrogene, *UTP14C*, associated with male fertility in man and mouse[4]–[6]. Retrogenes arise when fully or partially processed mRNAs are reverse transcribed into double stranded DNA and this DNA copy is inserted into the genomic DNA present in the chromosomes. If the copy is expressed as a messenger RNA and translated into a protein the new gene is known as a functional retrogene. The gene from which the retrogene originated is termed the progenitor gene. In humans *UTP14C* is located on chromosome 13 and originated as a reverse-transcribed copy of a gene located on the X chromosome, *UTP14A*. It has recently been established that a disproportionate number of functional autosomal retrogenes have originated from the X chromosome and acquired male germ-line specific function[7]–[9]. Several different hypotheses have been proposed to explain this evolutionary phenomenon[10], nevertheless the most compelling is that of compensation for transcriptional inactivation of the sex chromosomes that occurs in mammals when the sex bodies form during spermatogenesis[11]. During gametogenesis in the male the sex chromosomes pair via a short pseudoautosomal region during early prophase I and condense to form a macrochromatin body known as the sex, or XY, body[11]. Unlike the autosomal chromosomes that form homologous pairs known as synaptonemal complexes and remain transcriptionally active throughout meiosis, the sex chromosomes are transcriptionally silenced (meiotic sex chromosome inactivation) from the time of sex body formation until meiosis is competed and haploid spermatids form[12]. The X chromosome contains a large number of housekeeping genes essential for normal cell function and survival. Thus silencing of the X chromosome during male meiosis may have some metabolic disadvantage for later stages of spermatogenesis. Retrotransposition of X linked housekeeping genes to the autosomes, with subsequent acquisition of testis specific expression, is one mechanism by which such metabolic disadvantage can be corrected during spermatogenesis without disrupting normal function in somatic tissue[13]. In man there are at least eleven such autosomal retrogenes that have retrotransposed off the X chromosome and play an important role in maintaining efficient spermatogenesis[10].

Using endpoint RT-PCR to screen cDNAs prepared from a comprehensive panel of human tissues we found that *UTP14C* was expressed not only in the male testis but also in the female ovary and no other tissue[6]. The unexpected expression of *UTP14C* in the human ovary prompted us to explore whether or not *UTP14C* was also expressed in ovarian cancers. To determine if expression of testis specific retrogenes in normal ovaries and ovarian cancers was a general phenomenon in humans we also tested for the expression of four other retrogenes (*PGK2*, *RPL10L*, *RPL39L* and *UBL4B*) that were previously thought to be expressed exclusively during spermatogenesis in the male.

Here, we present the results of our screen for the expression of the testis specific retrogenes in normal ovarian tissue and a panel of ovarian cancers. We propose that a pattern of testis-like transcriptional regulation that results in retrogene expression frequently occurs in ovarian cancers and could contribute to the pathogenesis of this disease. In addition the unique ovarian and ovarian-cancer specific expression of retrogene encoded products may provide new understanding of gene expression in the female and lead to the identification of new diagnostic and/or therapeutic targets for ovarian cancer treatment.

## Materials and Methods

### Sources of Tissue Samples and RNA

Samples of pre- and post- menopausal ovaries were prospectively collected during clinical-indicated surgeries performed at Baylor College of Medicine affiliated hospitals by gynaecological surgeons. In addition samples of flash-frozen ovarian cancers were obtained from the Multidisciplinary Gynaecologic Tumor Bank at The University of Texas M.D. Anderson Cancer Center (MDACC, Houston, Texas), human total testis and ovarian RNA was purchased from Panomics (Fremont, California, USA. Product #NA2007) and the human RNA panel was purchased from Clontec (Clontech Laboratories Inc., Mountain View California USA. Master Panel II, Product #636643).

All collection was done under the approval from the Institutional Review Board at the Baylor College of Medicine (Protocol H-14372). Permission to use human tissues was obtained from each patient by written consent using a form approved by the Institutional Review Board for Baylor College of Medicine and its affiliated institutions. Written consent was similarly obtained from patients donating tissue samples to the Multidisciplinary Gynecologic Tumor Bank at The University of Texas M.D. Anderson Cancer Center.

### RNA extraction and first strand synthesis

Prospectively collected tissue samples were placed into 10 ml of RNAlater (Ambion, Inc. Austin Texas, USA) and stored at −20°C until RNA extraction. RNA was extracted from weight tissue samples by thawing in TRIzol Reagent (Invitrogen Corp. Carlsbad California USA) followed by immediate homogenization using a Tissue-Tearor homogenizer (BioSpec Products, Inc. Bartlesville Oklahoma, USA). RNA was recovered using the TRIzol Reagent protocol. RNA was resuspended in nuclease free water and stored at −80°C. To produce cDNA 5 µg of RNA was treated with DNase (Turbo DNA-free kit, Ambion Inc.), precipitated and re-suspended to a concentration of 1 µg per µl. A RETROscript Kit (Ambion, Inc.) was used for first strand cDNA synthesis from 2 µg of RNA with a combination of oligo(dT)_18_ and random(dN)_15_ primers. After synthesis the 20 µl reaction mixtures were diluted to 150 µl with nuclease free water and stored at −20°.

### RT-PCR

Genes of interest were detected by end point PCR using gene specific primers (Table 1). Reaction volumes of 20 µl of diluted AccuPrime Super Mix II (Invitrogen, Corp.) containing 1 µl of cDNA and retrogene specific primers were heated to 94°C for 2 minutes then cycled 35 times through 94°C for 20 seconds, 58°C for 20 seconds, 68°C for 30 seconds, and kept at 15°C after the final cycle. PCR products were run out on 1.75% agarose gels containing ethidium bromide and bands visualized by UV illumination. Gels were photographed using a Kodak Gel Logic 200 Imaging System.

**Table 1:** Primers used for gene expression analysis by endpoint PCR.

Gene	Primer Name	Primer Sequence
*UTP 14a*	hUTP14x3F	TCCAGCTGCACACTAGAAGAAC
	hUTP14x3R	CTTTCAGAAACTCCATGGGCT
*GT8*	HumJSD5′test	CATTTGCTGGTTTCTGTTGGCCAG
*UTP14b*	HumJSDLend	GCCTTTGCTAAATTGCTGAATAAG
	HumJSD2R	AGGCTCATGGCTTGGAGAGAG
	HumJSD3R	TTGGCCATAATTGCCTTTGAC
*PGK1*	PGK1F	CACTGCTCACAGAGCCCACAGC
	PGK1R	CTGCTTAGCCCGAGTGACAGCCTC
*PGK2*	PGK2F	CACTGCACACCGCGCTCATAGT
	PGK2R	TAGCCTTGCTTGAGCCACAACTTG
*RPL10*	RPL10F	AGGGTTCACATTGGCCAAGTT
	RPL10R	TAAGAGGGGGGCAGCACA
*RPL10L*	RPL10LF	GGGTCCACATTGGTCAAGTC
	RPL10LR	CCCAAGGAGACAGTACTGCC
*RPL39*	RPL39proF	CCTCCTCTTCCTTTCTCCGCCATC
	RPL39proR	GTTCATAACAGATTCAGAGAGG
*RPL39L*	RPL39LF S2	TGGATTCAGATGAAACCTGGT
	RPL39LR S2	ATCCACCCTACTAGCACAGAGC
*UBL4A*	UBL4AF	CAGCTGACGGTGAAGGCGCTGCA
	UBL4AR	GTTTGACCACTAGGTTGAGCTTG
*UBL4B*	UBL4BF	TTCCTCACAGTCAAGCTGCTCCT
	UBL4BR	GCTGCATGATGACATTGATAGAG

## Results and Discussion

**Figure 1:**
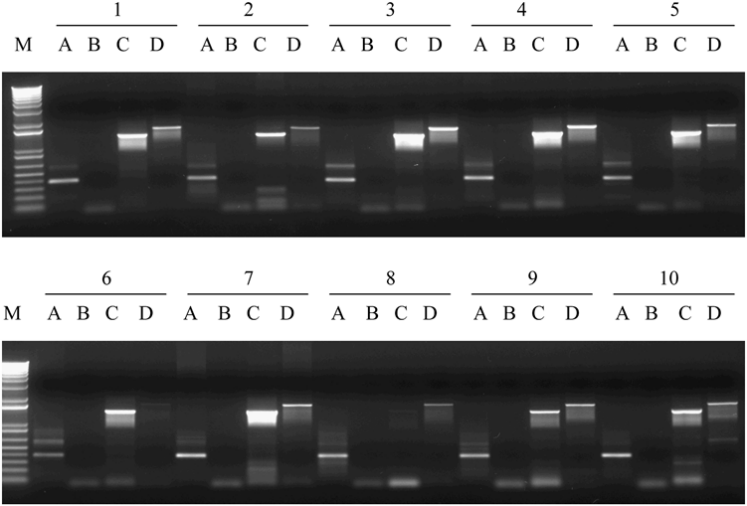
RT-PCR of cDNA samples from ten Papillary Serous ovarian cancers with primers specific to *UTP14A*, *UTP14C* and *GT8*. Lane A shows the product specific to *UTP14A*. Lane B shows the reaction product from a primer pair specific to *GT8* (5′ end) and *UTP14C* (3′ end), these two genes overlap on human chromosome 1 and the absence of a PCR product demonstrates that transcripts from the two gene are exclusive. Lane C shows the *GT8* gene specific product and, lane D, the PCR product from *UTP14C*. Molecular weight marker was run in lane M.

**Figure 2:**
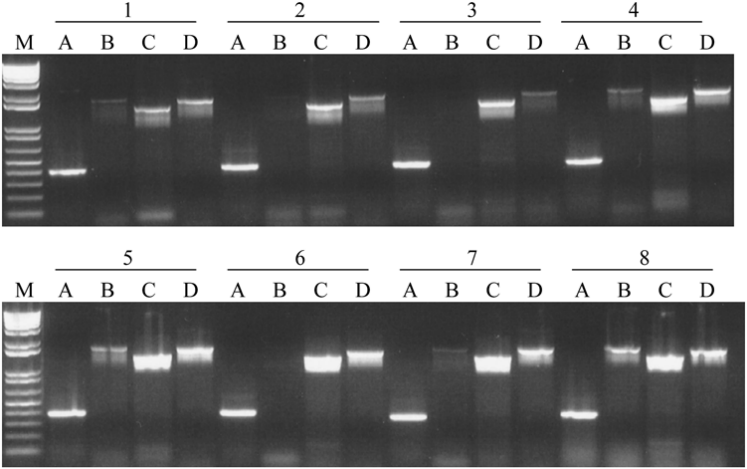
RT-PCR of cDNA from established ovarian cancer cell lines. Lane A: *UTP14A* specific product. Lane B: Product from primers common to both *UTP14C* and *GT8*. This primer combination fails to give a PCR product in ovarian or ovarian cancer tissue samples. Lane C: *GT8* specific RT-PCR product. Lane D: Product specific to *UTP14C*. Primer combinations are described in the text. Established ovarian cancer cell lines used were 1. 2774, 2. ES-2, 3. TOV112D, 4. OV90, 5. SKOV3, 6. TOV21G, 7. HEY, 8. OVCAR3, and M, molecular size marker.

As part of an ongoing program studying the role of genes crucial for spermatogenesis we previously reported the identification of a retrogene associated with male fertility in man and mouse[4]–[6]. In humans this retrogene, *UTP14C*, is located on chromosome 13 and originated as an intronless, reverse-transcribed copy of the X-linked gene, *UTP14A*. Although the function of *UTP14A* and *C* and their products is not known, analogy with the product from the yeast gene, *Utp14*, suggests that the human peptides, UTP14A and C, form part of the Small-Subunit (SSU) processome which is responsible for generating 18S rRNA from its precursor, the U3 snoRNA, in the nucleolus[14], [15]. Using endpoint RT-PCR to screen cDNAs prepared from a comprehensive panel of human tissues we found *UTP14C* to be expressed not only in the male testis, but also in the female ovary[6]. This unexpected expression of *UTP14C* in the human ovary prompted us to see if *UTP14C* was also expressed in ovarian cancers with the hope of identifying tissue specific markers selectively expressed in normal ovaries and ovarian cancers. End point RT-PCR was used to detect transcripts for *UTP14A* and *C* in primary ovarian cancers and established ovarian cancer cell lines. Determining the expression of *UTP14C* by RT-PCR is complicated by the fact that it is located within an actively transcribed host gene, *GT8*. *UTP14C* is the result of a retrotransposition event where an intronless DNA copy of the *UTP14A* coding sequence inserted immediately upstream of the coding region present in the terminal exon of *GT8* on chromosome 13. Subsequently *UTP14C* has acquired a promoter that drives its expression during spermatogenesis. Because the transcripts of *UTP14C* and *GT8* overlap the possibility of a single transcript containing open reading frames from both genes arises. To distinguish between the overlapping transcripts that are produced from the retrogene and its host, primer pairs were designed to specifically amplify either *UTP14C* or *GT8* [6] and detect any transcript that may contain both open reading frames. Fig. 1 shows the expression profiles of *UTP14A*, *UTP14C* and *GT8* in a representative sample of six different papillary serous ovarian cancers, the most common histological subtype of this malignancy, two clear cell and two endometrioid samples. *UTP14A*, the X chromosome linked progenitor gene from which *UTP14C* arose, was expressed in all of the cancer samples (lane A). Similarly, *GT8*, the host gene for *UTP14C*, was expressed in all samples (lane C). The spermatogenesis-associated retrogene, *UTP14C*, was expressed in all but one of the cancer samples (lane D). We also tested our samples using the combination of a 5′-primer specific to *GT8* (that is up stream of the UTP14C transcriptional start site) and 3′-primer specific to *UTP14C* (that is upstream of the *GT8* transcript)[6]. The absence of a PCR product using this primer pair confirms that a read-through mRNA, spanning both the *GT8* and *UTP14C* genes, is not produced and establishes the independence of the two overlapping transcripts (lane B). Similar results were obtained for all primer combinations when a panel of established ovarian cancer cell lines were tested under identical conditions (2774, ES-2, TOV112D, OV90, SKOV3, TOV21G, HEY and OVCAR3; Fig. 2). However, it should be noted that several of the cell line samples also showed the presence of RNA transcripts that spanned both the host gene (*GT8*) and *UTP14C* suggesting aberrant transcriptional regulation in some of the ovarian cancer cell lines. We have never observed transcription that spanned both genes in human tissue samples.

The high frequency with which *UTP14C* was expressed in our initial survey led us to explore the incidence of its expression in a large panel of ovarian cancers with different clinical histology. In aggregate, we found that that transcripts for *UTP14C* can be readily detected in 41/51 (80%) of papillary serous cancers, the most common form of cancer examined, and most other cancer subtypes. The frequency of *UTP14C* expression in a variety of ovarian cancer histological subtypes is shown in Table 2.

**Table 2:** The frequency of retrogene expression in a panel of ovarian cancer samples as well as pre- and post- menopausal ovary samples.

Cancer Histology	UTP14a	UTP14c	PGK1	PGK2	RPL10	RPL10L	RPL39	RPL39L	UBL4A	UBL4B
*Pap Serous*	50/51 (98%)	41/51 (80%)	48/51 (94%)	40/51 (78%)	45/51 (88%)	43/51 (84%)	50/51 (98%)	50/51 (98%)	50/51 (98%)	31/51 (61%)
*Mixed*	17/17 (100%)	10/17 (59%)	17/17 (100%)	13/17 (76%)	12/17 (71%)	13/17 (76%)	16/17 (94%)	17/17 (100%)	17/17 (100%)	5/17 (29%)
*Endometrioid*	9/9 (100%)	1/9 (11%)	7/9 (78%)	3/9 (33%)	7/9 (78%)	4/9 (78%)	9/9 (100%)	9/9 (100%)	7/9 (78%)	9/9 (100%)
*Clear Cell*	7/7 (100%)	1/7 (14%)	7/7 (100%)	4/7 (57%)	6/7 (86%)	4/7 (57%)	7/7 (100%)	7/7 (100%)	7/7 (100%)	2/7 (29%)
*MMMT*	1/1 (100%)	1/1 (100%)	1/1 (100%)	0/1 (0%)	1/1 (100%)	1/1 (100%)	1/1 (100%)	1/1 (100%)	1/1 (100%)	1/1 (100%)
*AGCT*	7/8 (88%)	2/8 (25%)	8/8 (100%)	4/8 (50%)	7/8 (88%)	5/8 (65%)	7/8 (88%)	7/8 (88%)	8/8 (100%)	6/8 (75%)
*Sex Cord*	1/1 (100%)	1/1 (100%)	1/1 (100%)	1/1 (100%)	1/1 (100%)	1/1 (100%)	1/1 (100%)	1/1 (100%)	1/1 (100%)	1/1 (100%)
*Transitional*	2/2 (100%)	1/2 (50%)	2/2 (100%)	2/2 (100%)	2/2 (100%)	2/2 (100%)	1/2 (50%)	2/2 (100%)	2/2 (100%)	2/2 (100%)
*Carcinoid*	1/1 (100%)	1/1 (100%)	1/1 (100%)	1/1 (100%)	1/1 (100%)	1/1 (100%)	1/1 (100%)	1/1 (100%)	1/1 (100%)	0/1 (0%)
*Immature Teratoma*	1/1 (100%)	0/1 (0%)	1/1 (100%)	1/1 (100%)	0/1 (0%)	1/1 (100%)	1/1 (100%)	1/1 (100%)	1/1 (100%)	0/1 (0%)
*Pre-Ovary*	5/5 (100%)	4/5 (80%)	5/5 (100%)	4/5 (80%)	5/5 (100%)	5/5 (100%)	5/5 (100%)	5/5 (100%)	5/5 (100%)	3/5 (60%)
*Post-Ovary*	9/9 (100%)	7/9 (78%)	9/9 (100%)	6/9 (67%)	9/9 (100%)	9/9 (100%)	9/9 (100%)	9/9 (100%)	9/9 (100%)	4/9 (44%)

Abbreviations: *MMMT*, Malignant Mixed Mullerian Tumor. *AGCT*, Adult Granulosa Cell Tumor. *Pre-*, Premenopausal. *Post-*, Postmenopausal.

To determine if activation of testis specific retrogenes in ovaries and ovarian cancers is a general phenomenon the expression of four other retrogenes, previously thought to be testis specific, was studied. The spermatogenesis associated retrogenes tested encoded for a variety of important cell functions: metabolic enzymes (*PGK2*)[13], ribosomal proteins (*RPL10L* and *RPL39L*)[16] and post translational protein modification (*UBL4B*)[17]. Primer pairs specific to the retrogenes and their X linked progenitors were used for molecular characterization of gene expression in the same panel of ovarian cancer tissues previously described.

**Figure 3:**
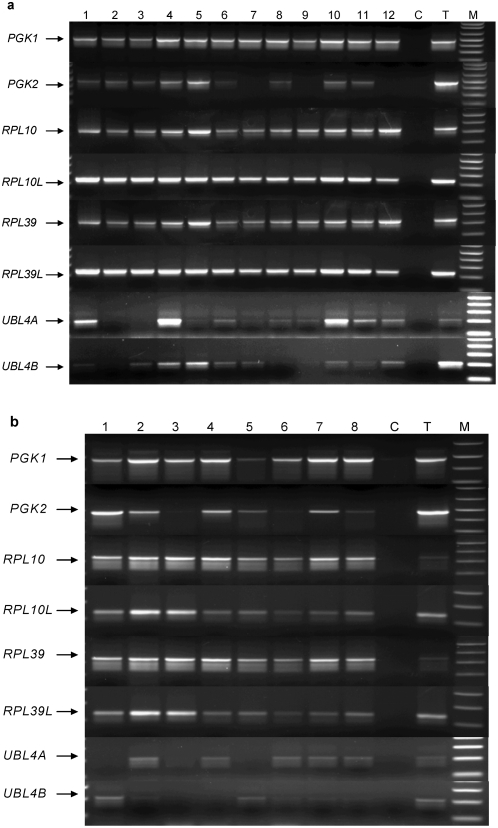
Expression of the testis specific retrogenes, and their X linked progenitors, in ovarian cancers and normal ovarian tissue. a. Retrogene expression in a panel of ovarian cancers was detected by RT-PCR. Samples and cancer histology were: 1–6. Papillary Serous, 7 & 8 Clear Cell, 9 & 10 Endometrioid, 11 pre-menopausal ovary, 12 post-menopausal ovary. Negative control C and marker M. b. Retrogene expression in normal ovarian tissue: 1–4. pre-menopausal ovaries, 5–8. post menopausal ovaries, negative control C and marker M.

In man, phosphoglycerate kinase is encoded by two genes, *PGK1* and *PGK2*. Phosphoglycerate kinase converts 1.3-diphosphoglycerate into 3-phosphoglycerate in the glycolysis pathway. This leads to the production of pyruvate from glucose and fructose[18]. *PGK1* is located on the X chromosome and is ubiquitously expressed whereas *PGK2*, a retrotransposed copy of *PGK1*, is located on chromosome 6 and shows a testis specific expression pattern[13]. Another retrotransposed copy of *PGK1* is present on chromosome 19 but it translates into a truncated protein and is probably inactive as no ESTs (Expressed Sequence Tags) could be found in the human database. Over expression of the PGK1 protein has been linked to paclitaxel resistance[19] and tumor angiogenesis[20]. In addition, PGK1 protein levels are elevated in serum from pancreatic duct adenocarcinoma patients[20], [21]. Thus, it appears that *PGK1* over expression may be of biological significance in some cancers. Detection of *PGK1* and *PGK2* in a representative panel of ovarian cancers, testis and pre and post-menopausal ovaries is shown in Fig. 3. Expression of *PGK2* was detected in all ovarian tumor types with the highest frequency in papillary serous (78%) and mixed histology (76%) tumors (Table 2). As expected for a housekeeping gene, *PGK1* was expressed in all tumor samples. However, due to the small sample size of the rare ovarian cancer subtypes the true frequency of *PGK2* expression in these tumors could not be estimated. It remains to be determined if *PGK2* expression is associated with tumor biology.

Mammalian ribosomes are made up of at least 80 ribosomal proteins[22] of which four are encoded on the X chromosome[23]. Three of these, *RPL10*, *RPL36A* and *RPL39*, have autosomal retrotransposed copies. Two are located on chromosome 14 (*RPL10L* and *RPL36AL*) and the third on chromosome 3 (*RPL39L*)[16]. *RPL36AL* is ubiquitously expressed whereas *RPL10L* and *RPL39L* have previously been reported to be expressed exclusively in the testis[16]. The *RPL10* is also a tumor suppressor gene and the protein it encodes is known as the tumor suppression protein QM[24]. Reduced *RPL10* expression is associated with increasing grade of prostatic adenocarcinoma[25]. Primers specific to *RPL10L* and *RPL39L* were used to screen for expression of these testis specific retrogenes in a panel of ovarian cancers (Table 2; Fig. 3). Transcripts from both retrogenes were detected in papillary serous, clear cell and endometroid samples. *RPL10L* was expressed in 76% of a large ovarian tumor panel with 84% expression in papillary serous cancers, 76% in tumors with mixed histology and 44% in endometroid tumors (Table 2). *RPL39L* was expressed at even higher frequency with 98% of papillary serous cancers containing transcript (Table 2). Expression in tumors with other histologies was 88% in AGCT (Adult Granulosa Cell Tumor) and 100% in all other subtypes tested.

**Figure 4:**
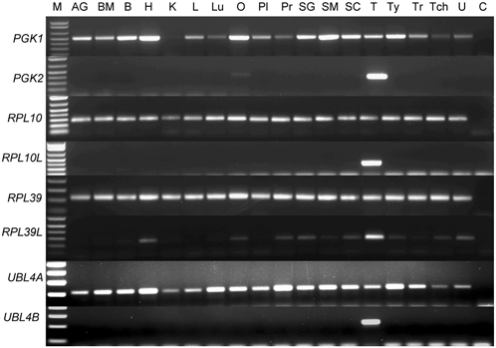
Tissue-specific expression patterns of *PGK1*, *PGK2*, *RPL10*, *RPL10L*, *RPL39*, *RPL39L*, *UBL4A* and *UBL4B*. Expression of the X linked progenitor genes *PGK1*, *RPL10*, *RPL39* and *UBL4A* is ubiquitous as would be expected for genes encoding essential cellular functions. In contrast expression of the spermatogenesis specific retrogenes, *PGK2*, *RPL10L*, *RPL39L* and *UBL4B*, is predominantly limited to the testis. Tissue key: AG, Adrenal Gland; BM, Bone Marrow; B, Brain; H, Heart; K, Kidney; L, Liver; Lu, Lung; O, Ovary; Pl, Placenta; Pr, Prostate; SG, Salivary Gland; SM, Skeletal Muscle; SC, Spinal Cord; T, Testis; Ty, Thyamus; Tr, Thyroid; Tch, Trachea; U, Uterus. M is the molecular size marker and C, is the primer only control reaction. The expression pattern for *UTP14A* and *UTP14C* has been previously published[5].

Although a direct role for the ribosomal proteins in cancer biology is yet to be established, it should be noted that *RPL39* expression is increased in breast cancer[26]. *RPL39L* expression has been reported in cervical cancer[27] as well as cell lines originating from breast, lung, prostate, colon, pancreatic and ovarian cancers[16], [20] suggesting that the retrogene *RPL39L* may have an important role in the biology of a large range of cancers. However, it should be noted that a screen for *RPL39L* expression in a human tissue RNA panel resulted in the detection of this retrogene in a large number of normal tissues suggesting that although *RPL39L* expression occurs predominantly in the testis it may not be testis specific (Fig. 4). Expression of the retrogenes *RPL10L* and *RPL39L* in cancers could possibly enhance ribosome production and/or translational activity. In testes, these retrogenes are expressed during spermatogenesis just before the rapid production of proteins that are required for sperm differentiation and maturation.

Ubiquitin like proteins (UBLs) are post translationally bound to a diverse group of other proteins thereby increasing the functional diversity of the encoded proteome. UBL modified proteins are known to influence the activity of a wide range of cell signaling pathways[28], [29]. *UBL4A* and *UBL4B* are recently identified genes that encode small ubiquitin like proteins[17]. In mouse, the X-linked *Ubl4A* is expressed in all tissue types, whereas the retrogene *Ubl4b* is reported to be expressed only in elongating spermatids during late stages of spermatogenesis in the testis[17]. Preliminary testing for *UBL4A* and *UBL4B* expression in a human RNA panel found that *UBL4A* was expressed in all tissue types tested. *UBL4B*, located on chromosome 1, was expressed only in the testis (Fig. 4) although later screening of a larger sample of pre- and post-menopausal ovaries demonstrated that *UBL4B* was expressed in normal ovarian tissues at low frequency (Table 2). Results of an initial screen for *UBL4B* activation in ovarian tumor samples is shown in Fig. 3. Table 2 shows the frequency with which *UBL4A* and *UBL4B* expression was detected in an extensive panel of ovarian tumor samples. As almost all cellular functions are affected by ubiquitination it is not surprising that alterations in ubiquitin and UBL activity have been implicated in the genesis of different human tumor types[30]. UBL modification by SUMO-1 (small ubiquitin-related modifier, also known as UBL1) has been directly linked to modification of the apoptosis pathway in ovarian tumors[31] and is known to play an important role in spermatogenesis[32].

Screening for retrogene expression in normal pre- and post- menopausal ovaries revealed that the retrogenes are not expressed in all women. The X linked progenitor gene is expressed in all cases (Table 2; Fig. 3) as would be expected for genes encoding essential gene functions that are ubiquitously expressed in the human body. However, retrogene expression in healthy ovarian tissue may be as high as 100% (*RPL10L* and *RPL39L*) of the samples tested or as low as 44% (*UBL4B*). This suggests that, for at least some of the retrogenes, expression in the ovary is limited to particular individuals within the general population. Most work on the spermatogenesis specific retrogenes has been done on the mouse model where ovarian expression of these genes is absent. Expression of these retrogenes in the human ovary may be a species specific phenomenon and may reflect an important biological difference between man and mouse. The retrogene and progenitor X gene specific primers were tested against a panel of RNAs from different human tissues to confirm the ubiquitous expression of the X linked progenitor and limited expression of the retrogene (Fig. 4). In most cases retrogene expression was limited to the gonads in man. Whether there is any relationship between expression of testis specific retrogenes in the human ovary and increased probability of cancer development is yet to be established.

Of special note is our observation that the X linked progenitor genes are not always expressed in all ovarian cancer samples. When this was the case the complimentary retrogene was always expressed suggesting that the retrogene was compensating for the loss of gene activity on the X chromosome in a manner similar to that which occurs when the X chromosome is transcriptionally silenced during male meiosis. The deletion of segments of the X chromosome in ovarian cancer cells and subsequent loss of heterozygosity is well documented[33]. Such deletions could result in the loss of genes which have retrotransposed copies. The loss of a copy of *PGK1* due to deletions within the X chromosome has been previously reported in a variety of ovarian cancer histological subtypes[34]. Such loss could be biologically significant if the deleted copy is from the active X chromosome or if both copies of a gene are transcriptionally active in normal cells.

The high frequency of spermatogenesis specific retrogene activation in ovarian cancers reported in this paper has important theoretical and practical implications for ovarian cancer treatment and biology. As these retrogenes are expressed only in the ovary and/or ovarian tumors and nowhere else in the female body the potential to develop tumor specific markers and therapies presents itself. Precedence for this is established by the expression of cancer/testis (CT) genes and antigens in a broad range of human tumors and is a phenomenon that has been recognized for some time[35], [36]. Expression of CT antigens in cancers was first reported with *MAGE-1* in 1991, even though at the time it was thought to be a gene expressed specifically in human melanoma tumors[37] and its expression in the testis was not recognized. Later reports added *MAGE-3* and *BAGE* to the list of CT antigens that are expressed in tumors but not expressed in normal tissues other than the testis[38], [39]. Identification of other CT antigens moved forward rapidly with the application of serological analysis of recombinant expression libraries (SEREX) technology[40]. To date at least forty four different CT antigens have been recognized[41]. The reason why genes, which encode proteins that are essential for spermatogenesis in the male or have a structural role in sperm, are expressed in cancers is not clear. The situation is complicated by the fact that the role of many of the CT genes in spermatogenesis is poorly understood[36]. However, this has not prevented the utilization of the CT antigens as biomarkers for developing novel cancer diagnostics[42] and/or targets for treatments such as immunotherapy[43]. One limiting factor in the practical application of CT antigens has been the low frequency with which the cancer/testis genes are expressed in any given tumor type. This restriction is not present in the case of spermatogenesis specific retrogene expression in ovarian cancers as these genes are expressed in a relatively high percentage of tumors.

An interesting feature of the previously recognized cancer/testis genes and antigens is their disproportionate association with the X chromosome[36]. The X chromosome and the genes it encodes appear to play a pivotal role in tumor genesis and development[44]. However, the relationship between tumor biology and spermatogenesis is less clear. In man it appears that the testis specific retrogenes are also be expressed in the ovary. However, the specific cell types within which expression occurs remains to be determined. Expression of these retrogenes may have a distinct metabolic advantage for ovarian tumor development as it may supplement gene products from the X chromosome, particularly in cases where the progenitor gene activity is down regulated by methylation[45] or lost due to deletion[34]. A link between metabolic events occurring during spermatogenesis and cancer development may be inferred from recent advances in the understanding of cancer biology. Of special note is the recently identified role of BRCA-1 in the development of breast and ovarian cancers[46]. The BRCA-1 protein has been shown to be associated with the inactive X chromosome in female somatic cells and, during spermatogenesis, is localized to the inactive X chromosome in pachytene spermatocytes during meiosis[47], [48]. This suggests a shared regulatory pathway. Further work is needed to determine the functional role of the spermatogenesis specific retrogene products in both spermatogenesis and cancer. Expression of genes and retrogenes in spermatogenic cells is driven by TATA-independent promoters. Polyadenylation sites are upstream of those utilized in somatic cells and there is a predominance of alternative pre-mRNA splicing[49]. The expression of testis specific genes and retrogenes suggests that cancer cells are able to utilize the transcriptional and RNA processing pathways normally active only during spermatogenesis in the male testis. Any structural identity shared by the mRNAs generated from the CT and other genes expressed in both cancers and testis needs to be determined. If spermatogenesis and tumor genesis share features of gene expression, regulation and RNA processing one could speculate that in long lived mammals cancer is a biological penalty of having separate sexes and the need for spermatogenesis in particular.
